# Sennoside A inhibits quorum sensing system to attenuate its regulated virulence and pathogenicity *via* targeting LasR in *Pseudomonas aeruginosa*

**DOI:** 10.3389/fmicb.2022.1042214

**Published:** 2022-11-03

**Authors:** Xiaofeng Han, Mengyue Nan, Xinyu Cai, Boling Qiao, Lin Chen, Lixin Shen

**Affiliations:** Key Laboratory of Resources Biology and Biotechnology in Western China, Ministry of Education, College of Life Science, Northwest University, Xi’an, China

**Keywords:** *Pseudomonas aeruginosa*, quorum sensing, sennoside A, inhibition, virulence, pathogenicity

## Abstract

*Pseudomonas aeruginosa* is an important opportunistic pathogen, and the emergence of drug resistance greatly increased the difficulty of treating its infection. Cell density-dependent quorum sensing (QS) system not only regulates the virulence but also associates with the drug resistance of *P. aeruginosa*. Screening for agents targeting QS to inhibit bacterial virulence and pathogenicity is considered a promising strategy to combat *P. aeruginosa* infection. In the present study, sennoside A was found to be able to inhibit the QS expression of *P. aeruginosa* at subinhibitory concentrations. The QS-regulated virulence factors, including protease, elastase, rhamnolipid, and pyocyanin, were also inhibited by sennoside A at both transcriptional and translational levels. Moreover, sennoside A could suppress the motility of twitching, swimming, and swarming as well as the biofilm formation, which is associated with the acute and chronic infections of *P. aeruginosa* in a dose-dependent manner. The attenuated pathogenicity of *P. aeruginosa* by sennoside A was further verified by Chinese cabbage, *Drosophila melanogaster*, and *Caenorhabditis elegans* infection analysis. Further study found that sennoside A might target the *las* system, mainly LasR, to interfere with QS. All the results indicate that sennoside A could inhibit the QS system to attenuate its regulated virulence and pathogenicity *via* mainly targeting LasR in *P. aeruginosa* and further research to identify its anti-QS activity for other Gram-negative bacteria is warranted.

## Introduction

*Pseudomonas aeruginosa* is a widespread important opportunistic pathogen that could cause hospital-acquired infection and severe complications, especially in patients with compromised immune systems, such as cystic fibrosis or burn patients (Stover et al., 2000; Driscoll et al., 2007; Karlberg et al., 2018). *P. aeruginosa* infections are very difficult to eradicate due to their intrinsic and acquired resistance to many antimicrobial agents used in clinical therapy (Levy and Marshall, 2004). Antibiotic abuse has driven a much more emergence of multiple-resistant *P. aeruginosa*, including those resistant to carbapenems (Basha et al., 2020). However, the development of new drugs has slowed down over the decades. The cure rate has decreased and mortality increased dramatically, resulting in worldwide public health and economic problems (Medina and Pieper, 2016). Therefore, it is urgent to explore alternative strategies for the treatment of *P. aeruginosa* infection.

Quorum sensing (QS) system is a cell density-dependent intercellular communication system, ubiquitously present in pathogenic bacteria (Norizan et al., 2013). Three interconnected QS systems, acyl homoserine lactone (AHL)-based *las* and *rhl* systems and quinolone-based *pqs* system, exist in *P. aeruginosa* (Li et al., 2020). *lasI* and *rhlI* encode the signaling molecules of *N*-3-oxo-dodecanoyl-l-homoserine lactone (3-oxo-C12-HSL) and *N*-butanoyl-L-homoserine lactone (C4-HSL), respectively. Transcriptional regulators of LasR and RhlR could bind with 3-oxo-C12-HSL and C4-HSL, respectively, to regulate the expression of their corresponding downstream genes, like *lasA, lasB, rhlAB, toxA*, and *aprA* (Whiteley and Greenberg, 2001; Schuster and Greenberg, 2007; Lee and Zhang, 2015). Similarly, *pqsA* initiates the synthesis of the signaling molecule of 2-heptyl-3-hydroxy-4-quinolone (PQS), which binds to the receptor PqsR to regulate the expression of the downstream genes, like *rhlAB, phzABCDEFG*, etc. (Deziel et al., 2004; Lee and Zhang, 2015). The three QS systems are interconnected with a multi-layered hierarchy, *las* system is at the top of the signal network and could activate the *rhl* and *pqs* systems (Wang et al., 2020); the *rhl* system could downregulate the *pqs* system, whereas *pqs* system could upregulate *rhl* system (Welsh and Blackwell, 2016).

*Pseudomonas aeruginosa* could cause severe acute and chronic infection in planktonic or biofilm states (Sankar Ganesh and Ravishankar Rai, 2018). The acute infection of *P. aeruginosa* is accompanied by the participation of many virulence factors. For instance, *lasA*-encoded protease and *lasB*-encoded elastase could destroy interstitial tissue and expand infection foci of the host (Stehling et al., 2008); *rhlA*-encoded rhamnolipid acts as a surfactant to stimulate motility of cells and biofilm formation (Davey et al., 2003; Caiazza et al., 2005; Tremblay et al., 2007); *phzA1*-encoded pyocyanin is a redox-active phenazine compound that could mediate tissue damage and necrosis of host during infection (Lau et al., 2004). Chronic infection is mainly associated with the formation of biofilms (Xu et al., 2013). The motility of swarming, swimming, and twitching mediates the movement and adhesion of cells to the infective site, initiating the acute and chronic infection of *P. aeruginosa* (Khan et al., 2020). The production of virulence factors, biofilm formation, and motility are all regulated by the QS system of *P. aeruginosa* (Adonizio et al., 2008). Therefore, inhibiting the QS system to relieve or suppress bacterial virulence and infectivity could be a promising strategy to tackle the infection caused by *P. aeruginosa*.

Sennoside A (SA), an anthraquinone compound, has been commonly used as a stimulant laxative to cure constipation (Le et al., 2021). Lots of physiological properties have also been reported for sennoside A, such as anti-obesity (Le et al., 2019), anti-cancer (Khan et al., 2020), hypoglycemic (Wei et al., 2020), and anti-neurodegenerative activities (Gao et al., 2021). In this study, sennoside A was found to be able to inhibit the QS and QS-regulated virulence features, including virulence factor production, biofilm formation, motility as well as pathogenicity of *P. aeruginosa* at subinhibitory concentrations. The probable target of sennoside A to anti-QS of *P. aeruginosa* was also searched by molecular docking and virulence gene expression analysis with *lux*-based QS quintuple mutants. Our study indicated that sennoside A could inhibit QS system of *P. aeruginosa* to attenuate its regulated virulence and pathogenicity by targeting the *las* system, mainly LasR.

## Materials and methods

### Bacterial strains, culture conditions, and reagents

The strains and plasmids used in this study are listed in Supplementary Table 1. Unless otherwise specified, bacteria strains were cultured in Luria-Bertani medium (LB, 1% NaCl, 1% peptone, and 0.5% yeast extract) at 37°C with shaking of 200 rpm. Sennoside A was purchased from Shanghai Yuanye Bio-Technology Co., Ltd. (Shanghai, China) and dissolved in dimethyl sulfoxide (DMSO).

### Monitoring bacterial growth and gene expression

The plasmid pMS402 carrying a promoter-less *luxCDABE* reporter gene cluster was used to construct promoter-*luxCDABE* reporter fusions of *lasI* and other QS-associated genes as described previously (Wang et al., 2021). Briefly, the promoter region of *lasI* was amplified by a polymerase chain reaction and ligated to pMS402 to obtain the plasmid pKD-*lasI*. Then, pKD-*lasI* was electroporated into PAO1 to get the *lux*-based reporter strain PAO1 (pKD-*lasI*). The *lux*-based reporter strains for the other genes were constructed similarly.

The gene expression was measured as the chemiluminescence produced by the *luxCDABE* operon, which is located in downstream of the gene promoter. Specifically, overnight cultures of the reporter strains were diluted to the appropriate cell density and cultivated for an additional 3 h. Then, 5 μl of the culture was inoculated into the parallel wells containing a total of 95 μl LB broth without or with sennoside A at different concentrations (50 or 100 μg/ml) in the black clear-bottom 96-well plate. A total of 50 μl of sterilized liquid paraffin was added to each well to prevent evaporation during the experiment. Luminescence (counts per second, CPS) as well as OD_600_ were measured every 30 min for 24 h by a Synergy H1 microplate reader (BioTeck, Winooski, VT, USA).

### Measurement of virulence factors

#### Protease

Protease activity was evaluated with 2% non-fat milk powder (Luo et al., 2017). In brief, *P. aeruginosa* PAO1 was cultured in the presence or absence of sennoside A at 37°C for 12 h. The supernatants were collected and then filtered by a syringe-driven filter of 0.22 μm, and 200 μl of filtered supernatant from the different groups were added to 800 μl phosphate buffer (pH = 7.8) containing 2% non-fat milk powder. The mixture was incubated at 37°C for 30 min to determine the absorbance at 440 nm.

#### Elastase

Elastase activity was estimated by the elastin Congo red assay (Kessler and Safrin, 2014). The supernatants were prepared similarly to the protease analysis. For each sample, 200 μl of filtered supernatant was added to 800 μl of reaction buffer (1 mM CaCl_2_, 0.1 M Tris–HCl, pH = 7.0) containing 5 mg of Elastin-Congo Red (Sigma, Saint Louis, MO, USA) and incubated at 37°C with a shaking of 200 rpm for 3 h. Elastase activity was determined by measuring the absorbance of the Congo red solution at OD_495 *nm*_.

#### Rhamnolipid

Secretion of rhamnolipid was evaluated using a cetyl trimethyl ammonium bromide (CTAB)-methylene blue (MB) plate (Jadhav et al., 2011). Briefly, overnight cultures of PAO1 were diluted to the appropriate cell density and then 2 μl of the diluted cultures were inoculated on the center of the CTAB-MB plate (0.7 g/L KH_2_PO_4_, 0.9 g/L Na_2_HPO_4_, 2 g/L NaNO_3_, 0.4 g/L MgSO_4_, 0.1 g/L CaCl_2_, 0.16 mg/L FeCl_3_, 1.5 mg/L ZnSO_4_, 0.15 mg/L CuSO_4_, 1.5 mg/L MnSO_4_, 1.5 mg/L H_3_BO_3_, 20 g/L glycerol, 0.2 g/L CTAB, 5 mg/L MB, and agar 15 g/L) with different concentrations of sennoside A. The plates were incubated at 37°C for 3 days and rhamnolipid production was confirmed by the blue halos around the colonies.

#### Pyocyanin

Pyocyanin was extracted and determined as described by Essar et al. (1990). Briefly, 50 μl of PAO1 overnight culture was inoculated to 5 ml *Pseudomonas* broth (0.14% MgCl_2_, 1% K_2_SO_4_, and 2% peptone, pH = 7.2) containing 0, 50, and 100 μg/ml of sennoside A and then cultivated at 37°C for 16 h. After centrifugation at 8,000 rpm, 5 ml of supernatant was mixed with 3 ml of chloroform thoroughly and then the chloroform phase was transferred to a fresh tube and mixed with 1 ml of 0.2 N HCl. Pyocyanin was quantified by determining the absorbance of the upper phase at OD_520 *nm*_.

### Quantification of biofilm formation

Biofilm formation was measured using the crystal violet method (He et al., 2014). Briefly, 30 μl of PAO1 overnight culture was inoculated into the wells containing 3 ml LB broth with sennoside A at different concentrations of 0, 50, and 100 μg/ml in the 12-well polystyrene plate. The plate was cultivated at 37°C for 24 h with a static system to form biofilms. Following the removal of the supernatant and non-adherent cells, phosphate-buffered saline was used to gently rinse the wells three times and then 3.5 ml of 1% crystal violet was added into the wells for 15 min staining. The attached crystal violet was washed with anhydrous ethanol and its absorbance was measured at 595 nm to quantify biofilm formation.

### Motility detection

The motility of swimming, swarming, and twitching of PAO1 was analyzed according to the previous report (Wang et al., 2021). For the swimming and swarming assay, 2 μl of log-phase PAO1 culture was spot inoculated onto the swimming agar plates (1% peptone, 0.5% NaCl, and 0.3% agar; pH 7.2) and swarming agar plates (0.8% nutrient broth, 0.5% glucose, and 0.5% agar; pH 7.2), which contained different concentrations of sennoside A, i.e., 0, 50, and 100 μg/ml. Twitching motility was determined by subsurface stab assays with 1% LB agar plates containing different concentrations of sennoside A. All the plates were incubated at 37°C for 24 h and the motility was investigated.

### Pathogenicity assay

Chinese cabbage, *Drosophila melanogaster*, and *Caenorhabditis elegans* infection models were used to evaluate the anti-infection activity of sennoside A against *P. aeruginosa*.

#### Chinese cabbage infection model

The cabbage infection analysis was performed according to the previous report with slight modifications (Starkey and Rahme, 2009). Briefly, 50 μl of PAO1 overnight culture was inoculated to 5 ml LB broth with or without sennoside A and then cultured at 37°C for 12 h. After centrifugation, cells were rinsed and then resuspended in 10 mM MgSO_4_ to a final concentration of 10^7^ colony-forming unit (CFU)/ml. A total of 10 μl of resuspension from the different groups was taken and injected into the cabbage stems that have been sterilized with 0.1% hydrogen peroxide. The cabbage stems were placed in Petri dishes with filter paper impregnated with 10 mM MgSO_4_. All the samples were incubated at 30°C for 6 days and the rotten area was determined.

#### *Drosophila melanogaster* infection model

The fruit fly feeding assay was carried out as described previously (Chugani et al., 2001). PAO1 was cultivated in LB broth containing sennoside A at 0, 50, and 100 μg/ml concentrations, respectively, for 12 h. The pellets from 1.5 ml cultures were suspended in 100 ml of 5% sucrose. Then, 200 μl of the resuspensions were spotted on the sterile filter disk that completely covered the surface of the sucrose agar (5% sucrose and 1% agar) contained in the flasks. Twenty 3- to 5-day-old male fruit flies that have been starved for 3 h were placed into each of the prepared flasks. The number of live fruit flies was counted every 24 h for 10 days to calculate the survival rate of the fruit flies.

#### *Caenorhabditis elegans* infection model

The *C. elegans* infection analysis was performed as reported previously (Sarabhai et al., 2013). Briefly, 100 μl of overnight PAO1 culture was spread evenly on the nematode culture medium (NGM) plates (0.3% NaCl, 0.25% tryptone, 1 mM MgSO_4_, 1 mM CaCl_2_, 5 μg/ml cholesterol, 100 μg/ml 5-Fluorouracil, and 2% agar) with different concentration of sennoside A and cultivated at 37°C for 12 h to form a bacterial lawn. Plates coated with *Escherichia coli* OP50 served as negative controls. Synchronized *C. elegans* N2 hermaphrodite worms at the L4 phase were seeded onto the prepared NGM plates. The plates were incubated at 20°C and the survival rate of nematodes was recorded every 12 h for 5 days.

### Molecular docking

Molecular docking was performed according to the previous report (Dai et al., 2019). The 3D structure of the proteins, LasR, LasI, PqsA, and PqsR, were downloaded from the online Protein Data Bank.^1^ The structure of sennoside A was obtained from the online website.^2^ The binding interaction of sennoside A with the QS-associated proteins in *P. aeruginosa* was evaluated by Auto Dock. The docking results were displayed using the PyMOL Molecular Graphics System (v. 2.3.4). The root mean square deviation (RMSD) values of each docking task based on the ligand sennoside A were calculated by the PyMOL Molecular Graphics System (v. 2.3.4).

### Statistical analysis

All the experiments were repeated three times and the values are presented as the mean ± SD. Statistical analysis was carried out by GraphPad Prism version 5.0.1 to determine significant differences (*p* < 0.05).

## Results

### Sennoside A decreases the expression of quorum sensing-associated genes in *Pseudomonas aeruginosa*

**FIGURE 1:**
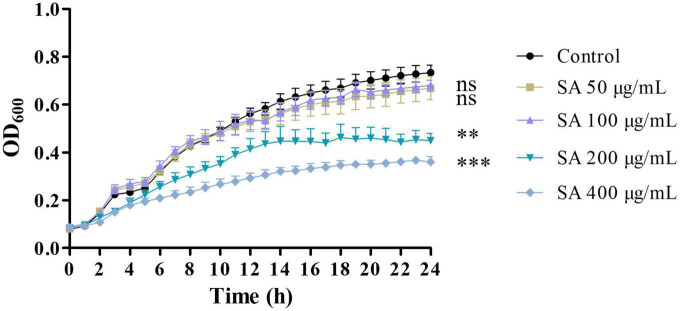
Effect of Sennoside A (SA) on the growth of PAO1. All data were expressed as mean ± SD values with three independent experiments performed in triplicate. ^ns^*p* > 0.05; ^**^p < 0.01; ^***^p < 0.001.

The effect of sennoside A on the growth of *P. aeruginosa* PAO1 was first assessed. As shown in Figure 1, sennoside A at concentrations of 50 and 100 μg/ml did not inhibit the growth of PAO1 compared with the control group with no sennoside A. Therefore, 50 and 100 μg/ml of sennoside A were used for the following studies.

**FIGURE 2:**
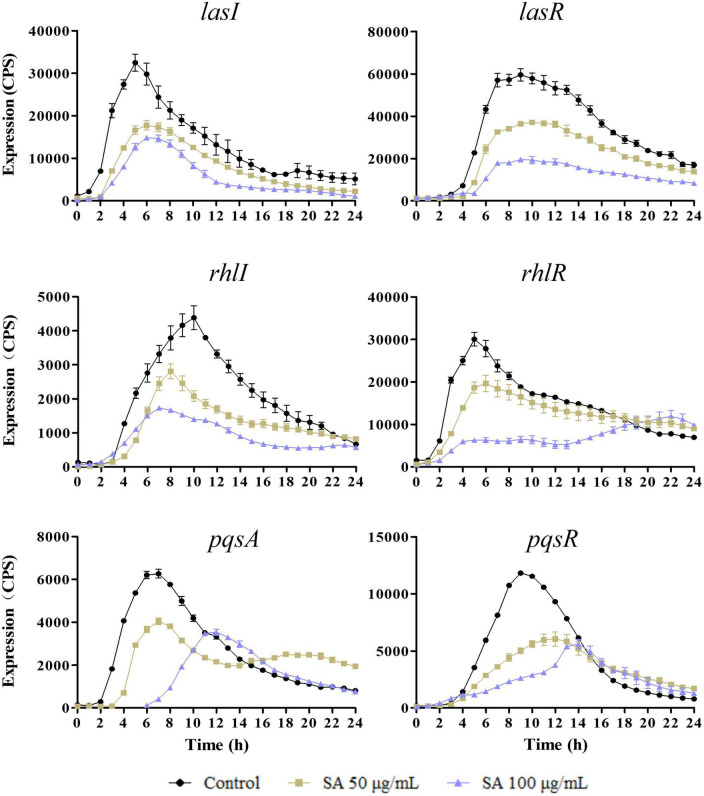
Sennoside A inhibits the expression of QS-regulated genes (*lasI, lasR, rhlI, rhlR, pqsA*, and *pqsR*) in PAO1. All data were expressed as mean ± SD values with three independent experiments performed in triplicate.

The expression of QS genes, *lasI, lasR, rhlI, rhlR, pqsA*, and *pqsR*, was determined in the presence of 0, 50, and 100 μg/ml sennoside A. As shown in Figure 2, the expression of examined genes was all decreased by sennoside A in a dose-dependent manner during the log phase. However, the expression of the autoinducer coding gene of *rhlI* whose peak was at 10 h was induced later than that of the receptor coding gene of *rhlR* whose peak was at 4–6 h, different from the general pathway of QS signaling. A possible reason is that RhlR might respond to alternative ligands in addition to its canonical C4-HSL, like PqsE (Mukherjee et al., 2017; Groleau et al., 2020), and it needs further study.

**FIGURE 3:**
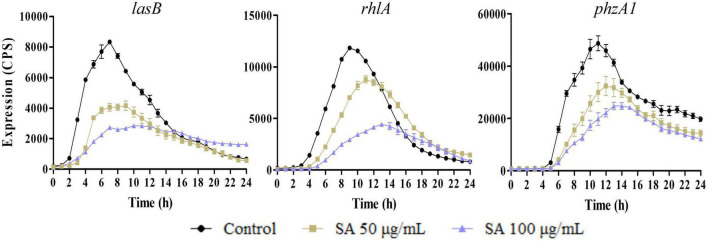
Inhibition of SA on the expression of virulence factor encoding genes (*lasB, rhlA*, and *phzA1*). All data were expressed as mean ± SD values with three independent experiments performed in triplicate.

Moreover, the influence of sennoside A on the expression of *lasB, rhlA*, and *phzA1*, which were the important virulence factor coding genes, and respectively, regulated by *las, rhl*, and *pqs* system, was also determined similarly. The results indicated that they were all downregulated by sennoside A in a dose-dependent manner compared with the control group (Figure 3).

### Sennoside A inhibits quorum sensing-controlled virulence in *Pseudomonas aeruginosa*

**FIGURE 4:**
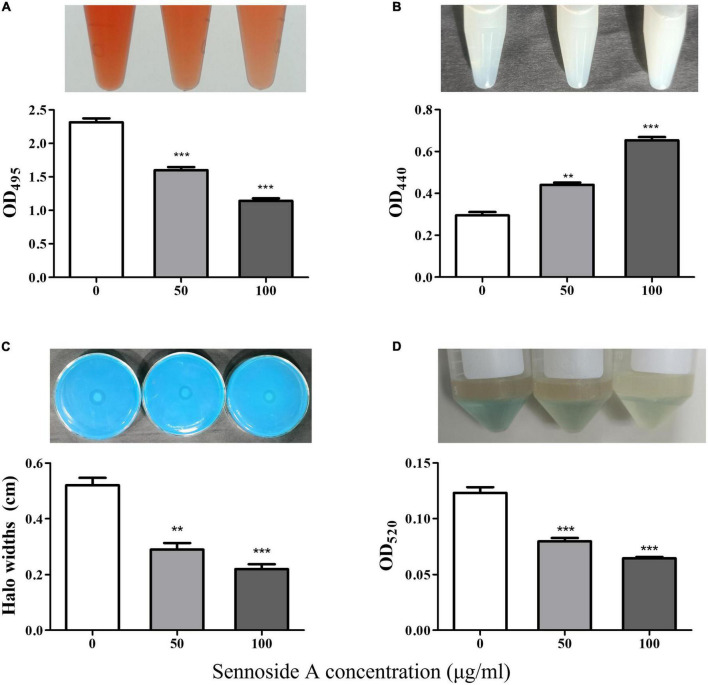
Inhibition effect of SA on the production of virulence factors in PAO1. **(A)** Protease; **(B)** elastase; **(C)** rhamnolipid; and **(D)** pyocyanin. All data were expressed as mean ± SD values with three independent experiments performed in triplicate. **p < 0.01; ***p < 0.001.

The production of the QS-controlled virulence factors in PAO1, including elastase, rhamnolipid, and pyocyanin, which were encoded by *lasB, rhlA*, and *phzA1*, respectively, was measured in an LB medium with and without sennoside A. As shown in Figure 4A, the absorbance at 495 nm was, respectively, decreased by 30.9 and 50.7% in the presence of 50 and 100 μg/ml sennoside A compared with the control group. The results indicated that sennoside A inhibits the production of elastase in a dose-dependent manner. Similarly, the production of other virulence factors of protease (Figure 4B), rhamnolipid (Figure 4C), and pyocyanin (Figure 4D) was also reduced significantly by sennoside A in a dose-dependent manner compared with the control group.

**FIGURE 5:**
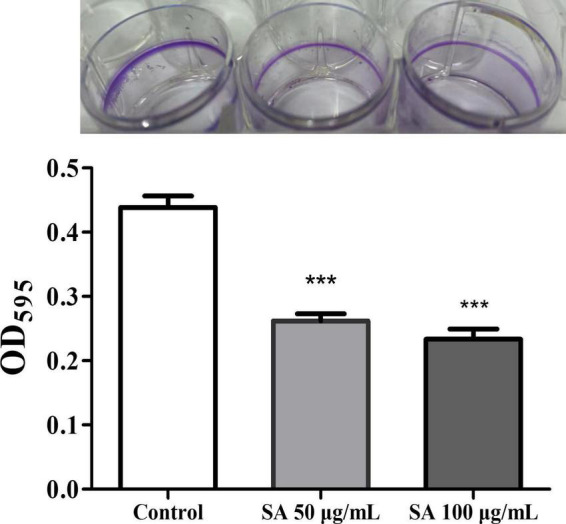
Impact of SA on the biofilm formation of PAO1. All data were expressed as mean ± SD values with three independent experiments performed in triplicate. ***p < 0.001.

**FIGURE 6:**
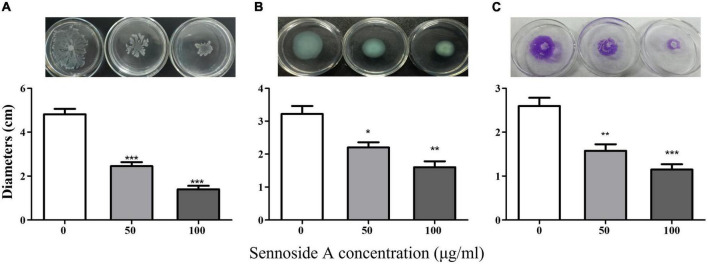
Effect of SA on the motility of PAO1. **(A)** Swimming; **(B)** swarming; and **(C)** twitching. All data were expressed as mean ± SD values with three independent experiments performed in triplicate. *p < 0.05; **p < 0.01; ***p < 0.001.

Biofilms and motility are additional crucial virulence features regulated by QS in *P. aeruginosa*. The impact of sennoside A on the biofilm formation and motility of swimming, swarming, and twitching was investigated in PAO1 accordingly. The results showed that sennoside A at 50 and 100 μg/ml reduced the biofilm formation by 40.3 and 46.7% (Figure 5), and inhibited swimming by 49.2 and 71%, swarming by 31.8 and 50.4%, and twitching by 39.4 and 55.8%, respectively, compared with the control (Figure 6).

### Sennoside A attenuates the pathogenicity of *Pseudomonas aeruginosa*

**FIGURE 7:**
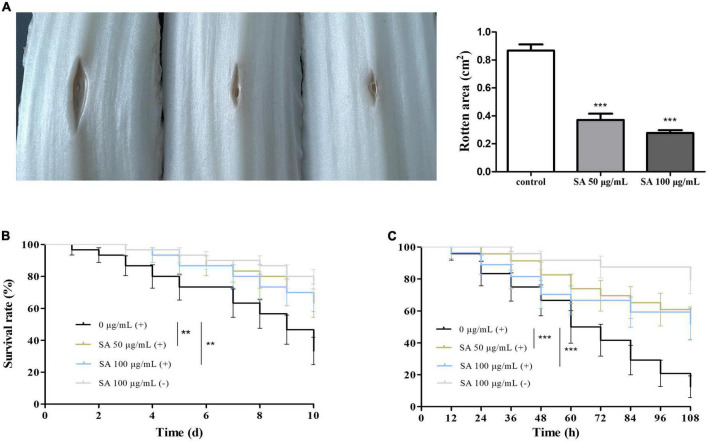
Effect of SA on the pathogenicity of PAO1. **(A)** Rotten area of Chinese cabbage; **(B)** survival rate of fruit flies; and **(C)** survival rate of nematodes. (+), with PAO1; (–), without PAO1. All data were expressed as mean ± SD values with three independent experiments performed in triplicate. **p < 0.01; ***p < 0.001.

Chinese cabbage, *D. melanogaster*, and *C. elegans* infection models were employed to evaluate whether sennoside A could attenuate the pathogenicity of *P. aeruginosa*. The results indicated that sennoside A could decrease the infection of PAO1 in Chinese cabbage because the rotten areas caused by PAO1 were reduced by 57.3 and 68.1%, respectively, in the presence of sennoside A at 50 and 100 μg/ml compared with that in absence of sennoside A (Figure 7A). In the *D. melanogaster* infection model, the 10-day survival rate of fruit flies was 33.3% in the control group while those in 50 and 100 μg/ml sennoside A treatment groups were increased to 63.3 and 66.7%, respectively, suggesting that sennoside A could decrease the mortality of the fruit files caused by PAO1 (Figure 7B). Similarly, the 108-h survival rate of infected nematodes was improved by nearly 40% under sennoside A treatments compared with the control (Figure 7C). All the results illustrated that sennoside A exerts a constructive impact on reducing the pathogenicity of *P. aeruginosa*.

### Molecular docking of sennoside A with quorum sensing-related proteins in *Pseudomonas aeruginosa*

**FIGURE 8:**
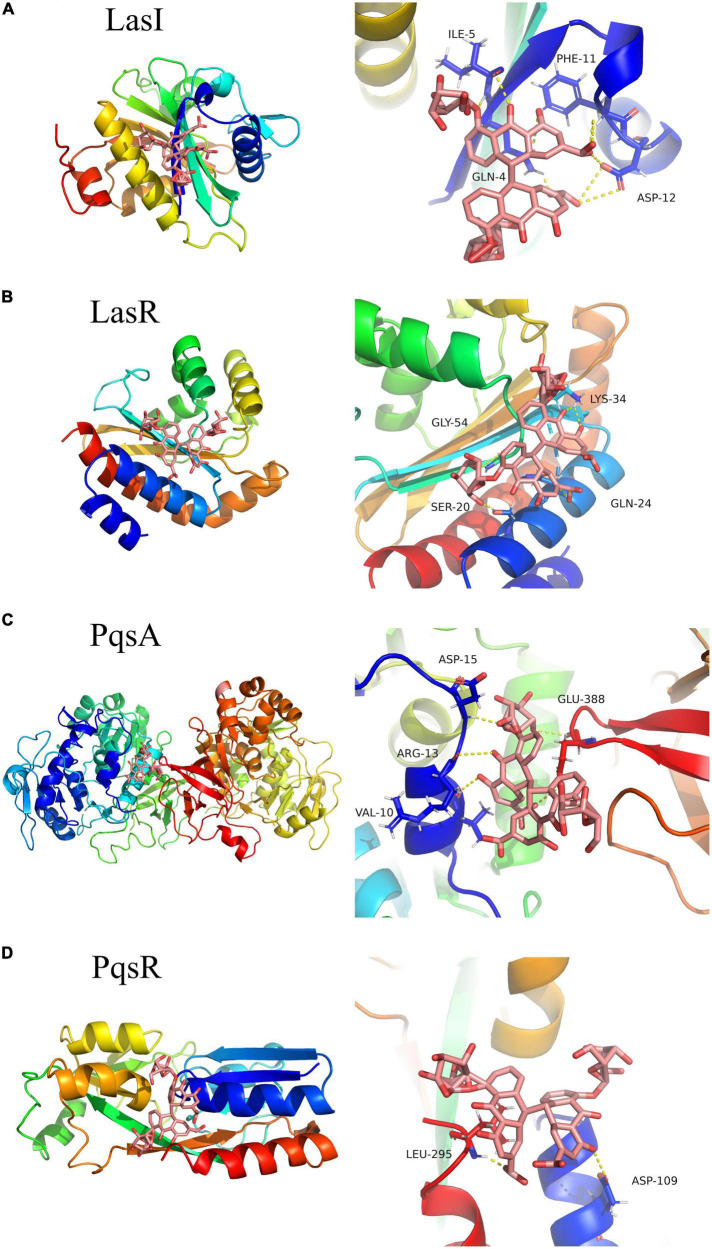
Three-dimensional **(left)** and 3D partial enlarged graphics **(right)** of molecular docking analysis for SA binding to QS-related proteins of *P. aeruginosa*. **(A)** LasI, PDBID: 1RO5; **(B)** LasR, PDBID: 6D6A; **(C)** PqsA, PDBID: 5OE6; and **(D)** PqsR, PDBID: 4JVD.

Molecular docking analysis was performed to explore the interaction of sennoside A with the QS-related proteins of LasI, LasR, PqsA, and PqsR in *P. aeruginosa*. RhlI and RhlR were not used for the analysis since the crystal structures were not available. The results of AutoDock analysis showed that sennoside A could form hydrogen bonds with Gln4, Ile5, Phe11, and Asp12 of LasI (Figure 8A), and the binding energy between sennoside A and LasI was −4.67 kCal/mol. RMSD values of sennoside A binding with LasI was 0.800 Å. Sennoside A binds to LasR with Ser20, Gln24, Lys34, and Gly54 residues by hydrogen bond interactions (Figure 8B), the binding energy was −5.85 kCal/mol, RMSD of sennoside A binding with LasR was 1.295 Å. Additionally, sennoside A forms hydrogen bonds with Val10, Arg13, Asp15, and Glu388 of PqsA (Figure 8C) and Asp109 and Leu295 of PqsR (Figure 8D). The binding energy of sennoside A to PqsA and PqsR was −4.47 and −4.97 kCal/mol, respectively, and RMSD of sennoside A binding with PqsA and PqsR was 0.958 and 1.890 Å, respectively. The results indicated that sennoside A might probably inhibit the QS system of *P. aeruginosa* by interacting with LasR since sennoside A has the highest binding scores with LasR. However, the interaction of sennoside A with RhlI/RhlR could not be excluded.

### Sennoside A impairs quorum sensing system of *Pseudomonas aeruginosa* by mainly targeting LasR

To further explore the target of sennoside A in inhibiting QS of *P. aeruginosa*, three quintuple mutants, PAO1 (Δ*lasI*Δ*rhlI*Δ*pqsA*Δ*rhlR*Δ*pqsR*) referred to as QM-1, PAO1 (Δ*lasI*Δ*rhlI*Δ*pqsA*Δ*lasR*Δ*pqsR*) referred to as QM-2 and PAO1 (Δ*lasI*Δ*rhlI*Δ*pqsA*Δ*lasR*Δ*rhlR*) referred to as QM-3, which separately contained only one kind of receptor coding gene of *lasR, rhlR*, or *pqsR*, were constructed and used. Since LasR and RhlR could upregulate the expression of *lasB*, and PqsR could upregulate the expression of *pqsA*, three strains of QM-1 (pKD-*lasB*), QM-2 (pKD-*lasB*), and QM-3 (pKD-*pqsA*) were further constructed. Then, the light production from QM-1 (pKD-*lasB*), QM-2 (pKD-*lasB*), and QM-3 (pKD-*pqsA*) were determined in the LB medium in presence of corresponding exogenous signaling molecules of 3-oxo-C12-HSL, C4-HSL or PQS with or without sennoside A.

**FIGURE 9:**
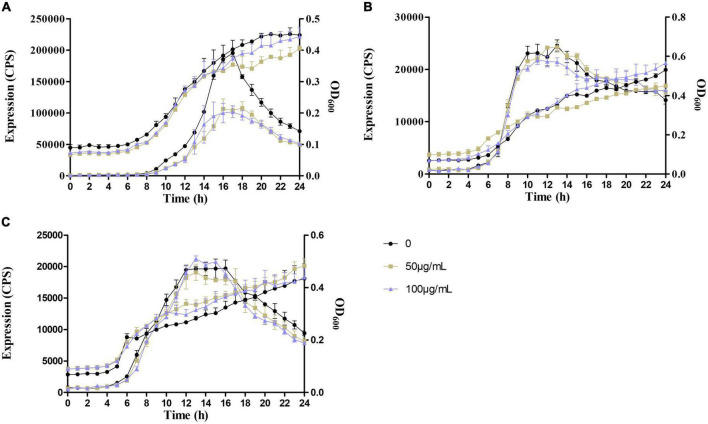
Effects of SA on the expression of *lasB* and *pqsA* in the QS quintuple mutants. **(A)** The expression of *lasB* in QM-1(pKD-*lasB*); **(B)** the expression of *lasB* in QM-2(pKD-*lasB*); and **(C)** the expression of *pqsA* in QM-3(pKD-*pqsA*). All data were expressed as mean ± SD values with three independent experiments performed in triplicate.

As a consequence, the expression of *lasB* in QM-1 (pKD-*lasB*) was reduced significantly by sennoside A in a dose-dependent manner, while the expression of *lasB* in QM-2 (pKD-*lasB*) and that of *pqsA* in QM-3 (pKD-*pqsA*) were not affected by sennoside A (Figure 9). Concurrently, sennoside A has no effect on the growth of QM-1 (pKD-*lasB*), QM-2 (pKD-*lasB*), and QM-3 (pKD-*pqsA*) (Figure 9). The results suggested that the inhibition of sennoside A on the QS requires the presence of the *las* system.

Furthermore, the *las*R knockout mutant of PAO1 (Δ*lasR*) was used to determine the pathogenicity in the presence and absence of sennoside A *via* the cabbage infection model. The result revealed that the attenuated pathogenicity of PAO1 by sennoside A was not observed in the mutant of PAO1 (Δ*lasR*) in which LasR was inactivated (Supplementary Figure 1). The above results indicated that sennoside A inhibits QS of *P. aeruginosa* mainly by interacting with LasR.

## Discussion

The emergence of bacterial resistance has led to great difficulty in infection treatment and increased mortality, causing a global public health crisis (Levy and Marshall, 2004). According to a meta-analysis, multidrug-resistant *P. aeruginosa* infection showed a >2-fold higher risk of mortality than infection caused by susceptible *P. aeruginosa* (Yang et al., 2018). QS is a gene regulation system that is widely present in bacteria and controls various physiological activities of bacteria, such as antibiotic resistance, virulence, and pathogenicity (Norizan et al., 2013). Inhibition of the QS system has been reported to be able to attenuate virulence and infectivity, and is thus considered a promising strategy to tackle infections caused by multidrug-resistant bacteria including *P. aeruginosa* (Kalia and Purohit, 2011). Numerous natural and synthetic compounds have been reported as QS inhibitors (QSIs). However, many of them are not suitable for clinical application due to their inherent cytotoxicity.

Sennoside A, an anthraquinone compound, has been used as a laxative for a long history in China. It has also shown some other pharmacology properties including anti-bacterial (Ram Avtar, 2012). According to the subacute and chronic toxicity studies, sennoside A showed no significant toxicity to rats (max. 20 mg/kg) and dogs (max. 500 mg/kg) (Mengs, 1988). In this study, sennoside A displayed an alternative physiological role as a QSI to attenuate the virulence and pathogenicity of *P. aeruginosa*.

First, sennoside A at subinhibitory concentrations could decrease the expression of QS-associated genes of *lasI, lasR, rhlI, rhlR, pqsA, pqsR, lasB, rhlA*, and *phzA1* in *P. aeruginosa* in a dose-dependent pattern (Figures 2, 3). Likewise, the production of the virulence factors, *lasA* encoded protease, *lasB* encoded elastase, *rhlA* encoded rhamnolipid, and *phzA1* encoded pyocyanin, was also inhibited similarly by sennoside A. All these virulence factors play important roles during the infection of *P. aeruginosa* (Figure 4).

Biofilms are a community of bacteria attached to biological and abiotic surfaces (Luo et al., 2017). They are responsible for chronic infection and can decrease the sensitivity of *P. aeruginosa* to drugs as much as 1,000-fold (Anwar et al., 1990). In multidrug resistance *P. aeruginosa* isolates, the biofilm-forming ability was significantly higher (Nassar et al., 2022). Motility is another important virulence feature associated with initial acute infection and chronic infection of *P. aeruginosa*. It is indispensable in surface perception and adhesion, biofilm formation, and resisting host immune responses of *P. aeruginosa*. *P. aeruginosa* performs different motility, flagella-driven swimming, type IV pili impelled twitching, and co-coordinated swarming of flagella and type IV pili flagella, in accordance with environmental conditions (Yeung et al., 2009; Burrows, 2012; Khan et al., 2020). In this study, sennoside A was found to enable to restrain biofilm formation (Figure 5) and reduce the motility of swimming, swarming, and twitching significantly of *P. aeruginosa* (Figure 6).

Moreover, sennoside A could attenuate the pathogenicity of *P. aeruginosa* obviously because the rotten area in the cabbage stems caused by PAO1 was reduced (Figure 7A) and the survival rate of fruit flies and nematodes were increased significantly in the presence of sennoside A compared with those of the control group (Figures 7B,C).

To explore the target of sennoside A in QS inhibition of *P. aeruginosa*, the expression of *lasB* and *pqsA* was determined in QM-1, QM-2, or QM-3, respectively, in presence of corresponding exogenous signaling molecules of 3-oxo-C12-HSL, C4-HSL, and PQS with or without sennoside A. Sennoside A could suppress the gene expression only in the presence of the *las* system but not the *rhl* and *pqs* systems (Figure 9). Additionally, the deletion of *lasR* could eradicate the inhibitory effect of sennoside A on the pathogenicity of *P. aeruginosa* (Supplementary Figure 1). Together with the results derived from the molecular docking analysis (Figure 8), it could be concluded that LasR might be the essential target for the inhibition of sennoside A on QS-mediated virulence and pathogenicity of *P. aeruginosa*.

Moreover, the QS inhibition capacity of sennoside A on Gram-positive *Staphylococcus aureus* and Gram-negative *Acinetobacter baumannii* was further investigated by using a Chinese cabbage infection model. Interestingly, sennoside A could reduce the rotten area in the cabbage stems infected by *A. baumannii* but not those infected by *S. aureus* (Supplementary Figure 2), suggesting that sennoside A might show an inhibitive effect on QS of Gram-negative bacteria, but not for Gram-positive bacteria. *P. aeruginosa* and *A. baumannii* mainly use AHLs for cellular communication, whereas *S. aureus* uses autoinducing peptides as QS signaling molecules (Monnet and Gardan, 2015; Sun et al., 2021). An AHL-dependent QS system might be necessary for sennoside A to attenuate the pathogenicity of pathogens.

Traditional Chinese herbal medicines have been widely used for disease treatment, suggesting their safety for human consumption. Identifying potential QSIs from Chinese herbs is considered a feasible approach and some importance has been gained. Extracts from *Herba patriniae* (Fu et al., 2017) and *Angelica dahurica* (Chong et al., 2018) have been reported to enable to reduce the virulence and pathogenicity of *P. aeruginosa*, but without active components and targets identified. Some compounds derived from Chinese herbs have also been reported as QSIs. Falcarindiol from *Notopterygium incisum* could inhibit the QS of *P. aeruginosa via* destabilizing LasR (Zhao et al., 2021). Crude extract of *Rheum palmatum* L. has been previously found to be able to inhibit the QS of *P. aeruginosa* in our lab. Sennoside A is one of the main components of *R. palmatum* L. (Le et al., 2021). Sennoside A could probably respond to the inhibition of the extracts on the QS of *P. aeruginosa*, at least partially.

## Conclusion

In summary, our results indicated that sennoside A could inhibit QS system of *P. aeruginosa* to attenuate its regulated virulence and pathogenicity *via* mainly targeting LasR, and further research to identify its anti-QS activity for other Gram-negative bacteria is warranted. Combining antibiotics with QSIs to synergistically treat bacterial infections would be an interesting and promising strategy.

## Data availability statement

The original contributions presented in this study are included in the article/Supplementary material, further inquiries can be directed to the corresponding author.

## Author contributions

LS conceived and designed the experiments. XH performed the experiments and wrote the manuscript. MN and XC analyzed the experimental data. LC and BQ made some revisions. All authors contributed to the article and approved the submitted version.

## Footnotes

1
https://www.rcsb.org/

2
https://zinc15.docking.org/
