# Supplementary figures and images for: Sennoside A inhibits quorum sensing system to attenuate its regulated virulence and pathogenicity via targeting LasR in Pseudomonas aeruginosa
Source: Front Microbiol. 2022 Nov 3;13:1042214. doi: 10.3389/fmicb.2022.1042214 (PMC9668863; doi:10.3389/fmicb.2022.1042214)

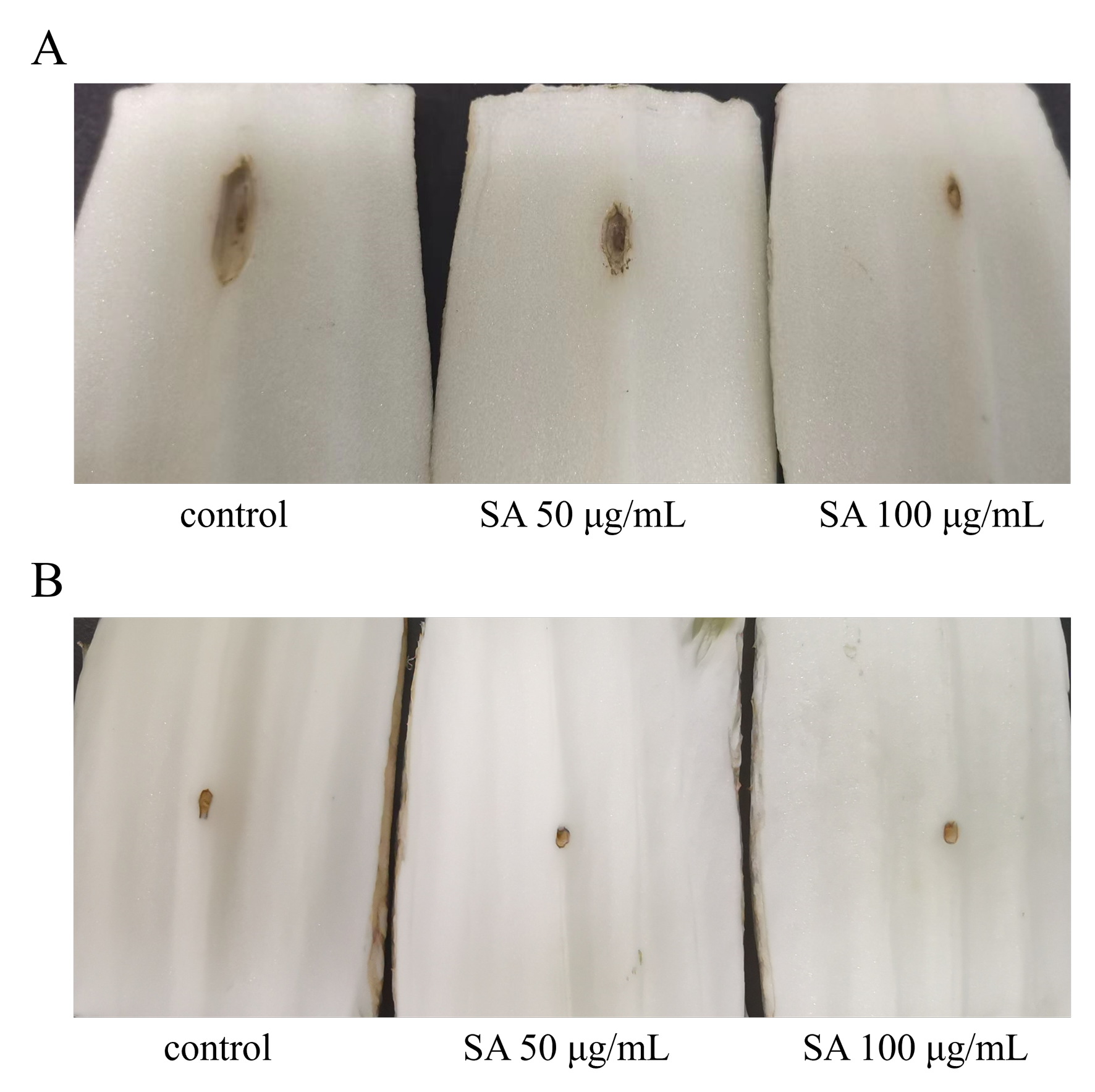

Supplement: Supplementary file 1 [file Image_1.TIF]

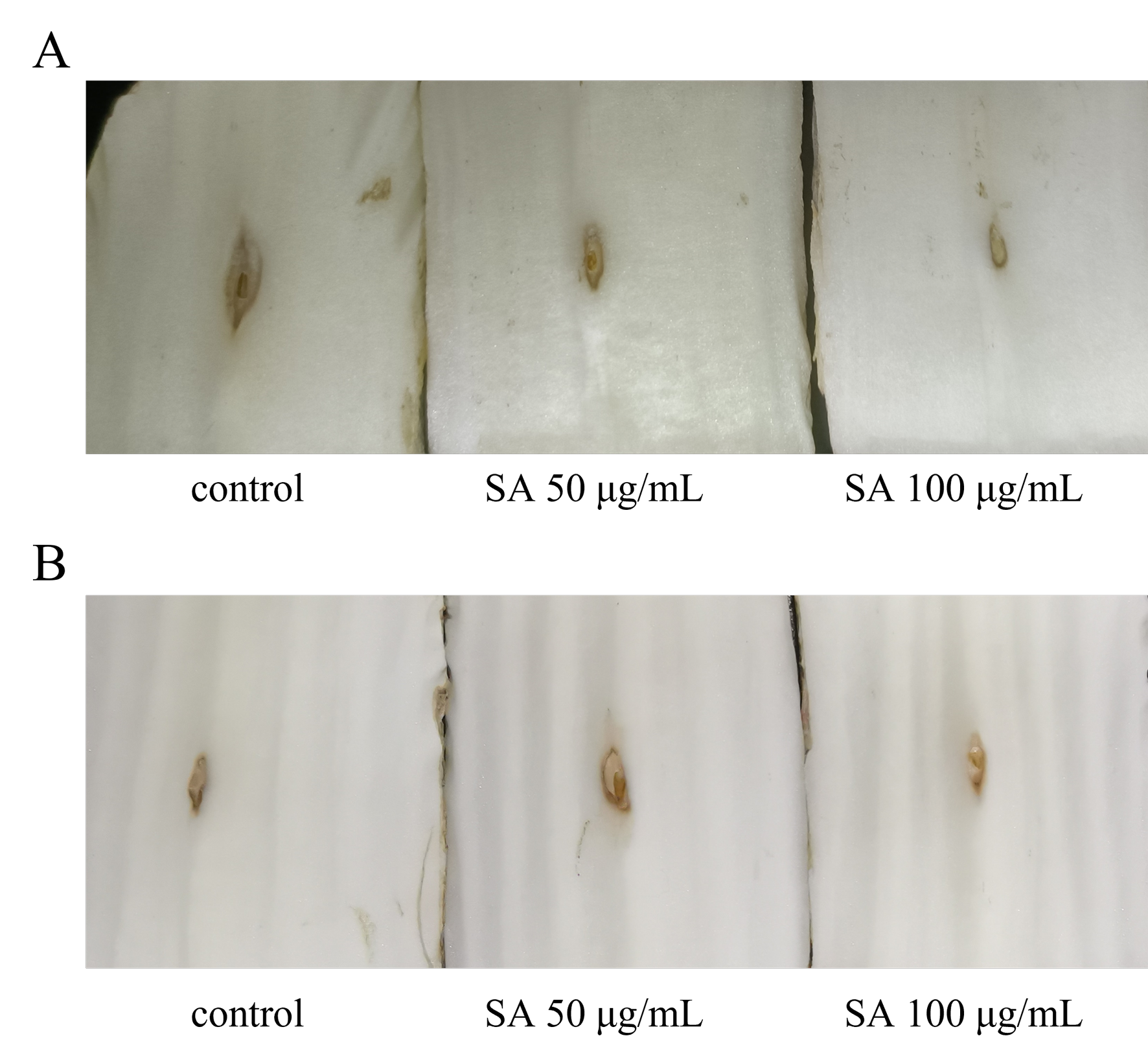

Supplement: Supplementary file 2 [file Image_2.TIF]
