# Supplementary material for: Sennoside A inhibits quorum sensing system to attenuate its regulated virulence and pathogenicity via targeting LasR in Pseudomonas aeruginosa
Source: Front Microbiol. 2022 Nov 3;13:1042214. doi: 10.3389/fmicb.2022.1042214 (PMC9668863; doi:10.3389/fmicb.2022.1042214)
Supplement: Supplementary file 3 [file Table_1.DOCX]

## Supplementary Table

**Supplementary Table 1.** Bacterial strains and plasmids used in this study.

| **Strains/Plasmids** | **Description** | **Source** |
| --- | --- | --- |
| **Strains** |  |  |
| *P. aeruginosa* PAO1 | Wild type strain | This lab |
| PAO1(Δ*lasR*) | *lasR* knocked out mutant of PAO1 | This lab |
| PAO1Δ*lasI*Δ*rhlI*Δ*pqsA*Δ*rhlR*Δ*pqsR* | PAO1 quintuple mutant with *lasI*, *rhlI*, *pqsA*, *rhlR* and *pqsR* knocked out | This lab |
| PAO1Δ*lasI*Δ*rhlI*Δ*pqsA*Δ*lasR*Δ*pqsR* | PAO1 quintuple mutant with *lasI*, *rhlI*, *pqsA*, *lasR* and *pqsR* knocked out | This lab |
| PAO1Δ*lasI*Δ*rhlI*Δ*pqsA*Δ*lasR*Δ*rhlR* | PAO1 quintuple mutant with *lasI*, *rhlI*, *pqsA*, *lasR* and *rhlR* knocked out | This lab |
| *E. coli OP50* | *E. coli* uracil-auxotrophic strain | This lab |
| *Staphylococcus aureus* | Clinical isolates strains | This lab |
| *Acinetobacter baumannii* | Clinical isolates strains | This lab |
| **Plasmids** |  |  |
| pMS402 | Expression reporter plasmid carrying the promoter-less *luxCDABE*; Kan^r^, Tmp^r^ | (Duan K. et al. 2003) |
| pKD-*lasI* | pMS402 containing *lasI* promoter region; Kan^r^, Tmp^r^ | (Duan K. and Surette M. G. 2007) |
| pKD-*lasR* | pMS402 containing *lasR* promoter region; Kan^r^, Tmp^r^ | (Duan K. and Surette M. G. 2007) |
| pKD-*rhlR* | pMS402 containing *rhlI* promoter region; Kan^r^, Tmp^r^ | (Duan K. and Surette M. G. 2007) |
| pKD-*rhlI* | pMS402 containing *rhlR* promoter region; Kan^r^, Tmp^r^ | (Duan K. and Surette M. G. 2007) |
| pKD-*pqsA* | pMS402 containing *pqsA* promoter region; Kan^r^, Tmp^r^ | (Liang H. et al. 2008) |
| pKD-*pqsR* | pMS402 containing *pqsR* promoter region; Kan^r^, Tmp^r^ | (Liang H. et al. 2008) |
| pKD-*lasB* | pMS402 containing *lasB* promoter region; Kan^r^, Tmp^r^ | (Liang H. et al. 2008) |
| pKD-*rhlA* | pMS402 containing *rhlA* promoter region; Kan^r^, Tmp^r^ | (Liang H. et al. 2008) |
| pKD-*phzA1* | pMS402 containing *phzA1* promoter region; Kan^r^, Tmp^r^ | (Liang H. et al. 2008) |

**References**

Duan, K., Dammel, C., Stein, J., Rabin, H., and Surette, M. G. (2003). Modulation of *Pseudomonas aeruginosa* gene expression by host microflora through interspecies communication. *Mol Microbiol*. 50:1477-1491. doi: 10.1046/j.1365-2958.2003.03803.x

Duan, K., and Surette, M. G. (2007). Environmental regulation of *Pseudomonas aeruginosa* PAO1 Las and Rhl quorum-sensing systems. *J Bacteriol*. 189:4827-4836. doi: 10.1128/JB.00043-07

Liang, H., Li, L., Dong, Z., Surette, M. G., and Duan, K. (2008). The YebC family protein PA0964 negatively regulates the *Pseudomonas aeruginosa* quinolone signal system and pyocyanin production. *J Bacteriol*. 190:6217-6227. doi: 10.1128/JB.00428-08

**Supplementary Figures**

**Supplementary Figure 1.** The inhibition of SA on the QS of *P. aeruginosa* via LasR. (A). Chinese cabbage infection model of PAO1; (B). Chinese cabbage infection model of PAO1(Δ*lasR*). Sennoside A decreases the pathogenicity of PAO1 but has no effect on the *lasR* mutant.

**Supplementary Figure 2.** Effects of sennoside A on the pathogenicity of other pathogens. (A). Chinese cabbage infection model of *Staphylococcus aureus*; (B). Chinese cabbage infection model of *Acinetobacter baumannii*. Sennoside A decreases the pathogenicity of *A. baumannii* but has no effect on *S. aureus.*
